# Supplementary material for: Are carbon emissions trading and green financial instruments synergistic? -Comprehensive quantitative research based on content analysis
Source: PLoS One. 2024 Mar 7;19(3):e0298601. doi: 10.1371/journal.pone.0298601 (PMC10919601; doi:10.1371/journal.pone.0298601)
Supplement: S1 Data — (ZIP) [file pone.0298601.s001.zip › Data/地级市Z_F工作报告.zip!/║■─╧/╥μ╤⌠╩╨/└┤╫╘╣1⁄2╓┌║┼í╛┬φ┐╦╩2╛▌═°í┐_2007.docx]

各位代表：

　　现在，我代表市人民政府，向大会作政府工作报告，请予审议，并请各位政协委员和其他列席人员提出意见。

一、2006年工作回顾

　　2006年是实施“十一五”规划的开局之年。全市各级各部门坚持以邓小平理论和“三个代表”重要思想为指导，全面落实科学发展观，开拓进取，扎实工作，较好地完成了市三届人大三次会议确定的各项任务，国民经济和社会发展取得了新的成就。

　　（一）经济发展速度加快，经济运行效益提高。预计全市完成地区生产总值335.1亿元，比上年增长12%，增幅比上年高0.7个百分点。其中第一产业增加值86.1亿元，增长6.8%；第二产业增加值103.1亿元，增长18%；第三产业增加值145.9亿元，增长11.7%。实施工业强市战略成效明显。完成工业增加值87.5亿元，增长19.2%。其中规模工业增加值60.5亿元，增长25.8%；经济效益综合指数135%，提高15个百分点。完成高新技术产业产值39亿元，增长25.8%。新培育湖南名牌产品10个。新农村建设健康推进，农业综合生产能力不断增强。农业产业化迈出新的步伐，优质稻米、林纸竹板、水产畜禽、棉麻纺织、油蔬茶果五大产业链基地面积突破1000万亩，新增省级龙头企业3家、市级龙头企业39家，新发展农民专业合作组织94个。全市完成春季造林117万亩，其中杨树造林101万亩，益阳被确立为全国唯一国家级现代林业建设示范市，安化、桃江被评为“湖南省林业十强县”。第三产业繁荣活跃。实现社会消费品零售总额130亿元，增长14%。商贸流通稳步发展，市场体系建设加快，“万村千乡市场工程”进展顺利。旅游业较快发展，接待游客人次和旅游总收入分别增长17.6%和15.3%。建筑业和房地产业发展进一步加快。金融业经营和监管水平提高；年末金融机构各项存款余额296.1亿元，各项贷款余额170.2亿元，比年初分别增长19.98%和6.6%。财税征管力度加大，财政收入较快增长，完成财政总收入19.02亿元，增长24.8%，其中完成地方财政收入11.59亿元，增长25.47%。

　　（二）改革开放不断深化，项目建设扎实推进。全市累计完成391家国有企业改革任务，占总数的92%，其中2006年完成40家。通过改革，一批企业焕发了生机与活力。农村综合改革全面推进。公务员管理机制不断完善，事业单位公开招聘制度全面实施。市直部门预算改革全面推行。文化体制改革等其他各项改革有序推进。招商引资成效显著。全市内联引资127.68亿元，增长47.2%；利用外资4131万美元，增长19.77%。开展了浙江招商活动周等招商活动。组织参加了首届中国中部贸易投资博览会，共签约项目86个，合同引资198.9亿元。引进了中核、中冶、中国昊华、浙江康超、西部矿业、紫金矿业、上海国之杰、唐人神等一批战略投资者。对外贸易取得新的突破，完成进出口总额1.82亿美元，比上年增长110%。固定资产投资快速增长。完成全社会固定资产投资130亿元，增长41.6%。修山电站两台机组并网发电。益阳电厂二期、东坪电站、株溪口电站、白竹洲电站、橡塑机械工业园、晶鑫光伏产业太阳能多晶硅、纳爱斯二期、博云高科二期、惠同三期、海纳百川、赤山大桥、麻河口大桥、马奎路、沅茅路、中心城区防洪工程、农村公路、城乡电网改造等重点项目正在抓紧建设。桃花江核电站、安化石煤综合发电、150万吨林浆纸等项目前期工作进展顺利。城市规划、建设和管理水平提高，中心城区扩容提质步伐加快，益阳大道竣工通车，市政公用设施日趋完善。全市投入小城镇建设资金7.5亿元，新增城镇人口3.3万人。

　　（三）社会事业全面发展，精神文明建设取得新成效。争取国家和省级科技项目41项，获省科技进步一等奖1项、二等奖2项、三等奖1项，评审奖励市科技进步奖28项，申请专利突破200项，其中发明专利占30%。自主创新能力不断增强，科技服务体系逐步完善。各级各类教育协调发展，素质教育全面推进，中心城区教育资源整合工作取得明显成效，湖南城市学院、益阳职业技术学院、益阳高级技工学校顺利搬迁，益阳卫校申办医专工作取得重大进展。公共卫生服务体系不断完善，疾病预防控制水平进一步提高，乡镇卫生院建设步伐加快，农村新型合作医疗制度全面推行，社区医疗服务逐步推进，医疗质量和医疗安全管理得到加强。食品药品监督工作力度加大。文化基础设施建设不断加强，群众文化生活更加丰富，全市有3个项目被评为湖南省首批非物质文化遗产代表作，文物保护工作取得新的成效。成功举办了第二届中国杨树产业可持续发展高峰论坛、首届桃花江国际生态文化旅游论坛和第二届洞庭渔火节。承办了第十九届世界杯羽毛球赛，参加省十运会取得金牌总数第五的好成绩，举办了市第八届大众运动会。计划生育打假治水和技术服务力度加大，部门综合治理更加得力，市级实现了进位争先的工作目标。环境保护和环保执法力度加大，造纸企业污染整治工作顺利推进。国土资源管理加强，建设项目用地得到保障，耕地保护目标完成，征地拆迁工作逐步规范。人防事业发展加快，城市信用社清整工作取得新成绩。新闻出版、广播电视、民政、民族、宗教、监察、审计、统计、价格管理、档案、外事、侨务、对台、地震、残联、科协、地方志、老龄、无线电管理、住房公积金等各项工作都有新的进步，国税、地税、电业、工商、质监、银行、保险、烟草、石油、盐务、电信、移动、联通、邮政、气象、水文等中央、省驻益单位的工作都取得了新的成绩。加强了落实科学发展观、构建社会主义和谐社会的宣传。认真开展了社会公德教育年活动，大力弘扬公民道德基本规范，特别是加强了以“八荣八耻”为主要内容的社会主义荣辱观教育。文明创建活动成果不断巩固，创星活动继续深入，乡风文明建设工程和“文明机关”、“和谐社区”等创建活动扎实开展，城乡文明程度进一步提高。深入开展了“扫黄打非”和网吧整治行动，文化市场和学校周边环境得到改善。

　　（四）民主法制建设得到加强，社会大局保持稳定。各级政府自觉接受人大及其常委会的法律监督、工作监督和政协的民主监督，严格执行人大及其常委会的决议、决定，认真听取民主党派、工商联、无党派人士的意见，及时办理人大代表的建议、批评、意见和政协委员的提案，共办理省市人大代表建议125件、省市政协委员提案170件，办复率100%。完成了第六次社区居委会换届选举工作，村民、居民自治和民主管理制度不断完善，基层民主政治建设进一步加强。“五五”普法全面启动。依法治市工作深入推进，《益阳市全面推进依法行政五年规划》全面实施，中心城区相对集中行政处罚权工作扎实开展。社会治安综合治理得到加强，开展了打黑除恶、打击“两抢一盗”、禁毒等专项行动和集中整治地下“六合彩”等赌博活动，“平安益阳”建设取得成效。正确处理人民内部矛盾，加大了信访问题和矛盾纠纷的调处力度，维稳工作机制不断完善。加强了安全隐患的排查和整改，安全生产工作取得成绩。坚持不懈地开展反腐败斗争，集中治理商业贿赂，纠风治乱工作深入推进。政务公开力度加大，政府管理创新稳步推进。积极开展国防教育和“双拥”活动，民兵预备役工作加强。工会、共青团、妇联等群众团体在三个文明建设中发挥了积极作用。

　　（五）人民生活继续改善，省市18件实事全面完成。全市城镇居民人均可支配收入9138元，增长10.8%；农民人均纯收入3386元，增长11.4%。全年转移输出农村富余劳动力96.4万人，实现劳务收入65.5亿元，分别增长9.4%和16.1%。就业再就业政策得到落实，社会保障体系建设不断加强。扶贫开发、移民工作和农村能源建设取得新的进展。城市低保、农村特困救助、城乡大病医疗救助、救灾救济等各项工作进一步规范和加强，特困群体基本生活得到保障。省市18件实事全面完成。其中茅草街大桥竣工通车；完成县到乡镇砼路面改造61公里、乡镇到村砼路面改造1368公里；完成农村中小学危房改造98所；新建城镇经济适用房26618平方米，城镇最低收入家庭廉租房竣工面积3100平方米；完成了中心城区10条小街小巷建设任务。

　　各位代表，过去的一年，我市主要经济指标在全省的位置出现了积极的变化，地区生产总值排名前移一位，规模工业增加值、全社会固定资产投资、财政总收入的增幅分别列全省第一位、第二位和第五位。这些变化，显示益阳经济社会已经步入加快发展的轨道。这是全市人民在中共益阳市委的正确领导下，求真务实、团结奋斗的结果，得益于市人大、市政协的全力支持和有效监督，得益于各级各部门的通力协作和紧密配合，得益于社会各界的倾心关注和大力支持。在此，我代表市人民政府，向奋战在全市各条战线的工人、农民、知识分子，向驻益人民解放军、武警部队、民兵预备役官兵和政法干警，向所有关心、支持益阳发展的朋友们表示衷心的感谢，并致以崇高的敬意！

　　在充分肯定成绩的同时，我们也清醒地看到，我市经济和社会发展中仍然存在一些不容忽视的困难和问题，主要是：经济总量依然偏小，经济结构不够合理；工业化水平低，经济增长方式粗放，完成节能、减排等约束性指标难度较大；社会保障覆盖面窄，保障能力不强，就业再就业形势仍然严峻；财政支出压力增加，县乡财政比较困难；改革和发展的深层次矛盾逐步显现，影响社会和谐稳定的因素依然较多；经济发展环境有待进一步优化，政府自身建设需要进一步加强。对于这些问题，我们将高度重视，并采取有效措施切实加以解决。

二、2007年工作的指导思想和奋斗目标

　　2007年是我市加快发展、后发赶超的重要一年。全球经济总体稳定，国际贸易和投资需求继续保持旺盛势头，世界范围产业转移加快。国内经济社会发展形势看好，产业布局孕育新的调整，沿海地区产业内移加速，泛珠三角区域经济合作加强，中部崛起战略顺利推进，新农村建设逐步深入。省内“3+ 5 ” 城市群建设的加速推进，为我市加快发展提供了新的机遇。益阳区位优势明显，优势产业发展加快，产业集群初现雏形，基础设施逐步完善，经济发展环境不断优化，加快发展具备较好基础。我们一定要以奋力拼搏、锐意进取的饱满激情，以开拓创新、敢为人先的无畏精神，抢抓机遇，乘势而上，努力开创我市经济社会发展的新局面。

　　2007年政府工作的指导思想是：坚持以科学发展观为指导，认真落实中央经济工作会议、省第九次党代会和市第四次党代会精神，紧紧围绕“十一五”期间我市综合实力跻身全省第二方阵的奋斗目标，突出推进新型工业化和建设社会主义新农村两个重点，继续大力实施工业强市、项目立市、开放活市、科教兴市、依法治市五大战略，切实抓好基础产业、基础设施和基础工作，加强节能降耗和生态环境保护，大力推进社会和谐，努力实现经济社会又好又快发展。国民经济和社会发展的主要预期目标是：地区生产总值增长12%；全社会固定资产投资增长30%；社会消费品零售总额增长14%；招商引资实际到位资金增长30%；财政总收入增长20%；城镇居民人均可支配收入增长9%，农民人均纯收入增长9%；新增城镇就业4.5万人，城镇登记失业率控制在4.5%以内；万元生产总值能耗下降4%；主要污染物排放总量减少4%；人口自然增长率控制在5‰以内。

　　实现上述目标，我们必须把握以下指导原则：

　　第一，坚定信心，奋力后发赶超。要充分看到我市经济社会发展的潜力，充分发挥资源优势、区位优势，振奋精神，坚定信心，紧紧抓住第一要务，切实增强后发赶超意识，聚精会神搞建设，一心一意谋发展，加快发展速度，提升发展质量，增强发展后劲。

　　第二，解放思想，致力改革创新。坚持以经济建设为中心，坚决冲破一切妨碍发展的思想观念，坚决改变一切束缚发展的做法和规定，坚决革除一切影响发展的体制弊端。用改革的办法、创新的思维促发展，放手让一切劳动、知识、技术、管理和资本的活力竞相迸发，让一切创造社会财富的源泉充分涌流。

　　第三，统筹兼顾，突出发展重点。既要注重发展的系统性、整体性，统筹推进经济、政治、文化和社会建设，又要紧紧抓住加快益阳发展的关键环节，实行重点突破。坚定不移地突出强力推进新型工业化进程；坚定不移地努力抓好社会主义新农村建设；坚定不移地抓好招商引资，特别是抓大项目、好项目和战略投资者的引进工作；要继续大力加强基础设施建设，努力做大做强中心城区，不断提升工业化、农业产业化和城镇化水平。

　　第四，以人为本，切实关注民生。要切实把实现好、维护好、发展好人民群众的根本利益作为政府一切工作的出发点和落脚点，以人民群众的满意度作为衡量各级政府工作的标准。要认真倾听民意，切实体察民情，有效激励民志。要千方百计扩大就业，着力强化社会保障，加快发展社会事业，注重社会公平公正，努力提高人民群众的生活质量和幸福感水平。

　　第五，优化环境，确保和谐稳定。坚持依法行政，切实转变政府职能，努力提高行政效能，大力推进政务公开，进一步优化发展环境。牢固树立和谐发展理念，促进经济发展和人口、资源、环境相协调，确保社会政治大局稳定，在和谐稳定的环境中推进改革发展，在改革发展中保持和谐稳定。

三、努力实现经济又好又快发展

　　（一）大力推进新型工业化

　　要把实施工业强市战略作为加快发展的重中之重。坚定不移地走科技含量高、经济效益好、资源消耗低、环境污染少、人力资源优势得到充分发挥的新型工业化道路，以工业的大发展带动全市经济的后发赶超。

　　着力培育产业集群。认真实施产业发展规划。加大对优势产业的投入，建立优势产业发展基金。通过抓好产业整合、产业深度广度开发、产业链的构建和产业服务平台建设，着力培育装备制造、电力、造纸、棉麻纺织、新材料、新能源、食品加工、有色冶炼、竹木加工等产业集群，集中扶持一批主业突出、竞争力强、带动作用大的优势骨干企业发展壮大。加快与长株潭的产业对接。坚持把培育大企业与发展中小企业结合起来，切实帮助中小企业解决融资等方面的困难，促进中小企业成长壮大，提高产业配套能力，促进产业集群的形成。加大对品牌经济的宣传、培育和扶植力度。

　　加快工业园区发展。加强对园区经济发展的目标管理考核工作。认真做好园区工业布局规划，强化园区体制机制创新，加强园区基础设施建设，提高园区的要素聚集和产业承载能力，培育工业经济的强势增长极。益阳高新技术产业开发区要大力发展新能源、新材料、生物医药、电子信息、先进制造等产业，着力打造新能源新材料基地。龙岭工业园、长春工业园要加快发展主导产业，实现与高新区的产业互补。桃江、沅江、南县、安化、大通湖经济开发区要发挥比较优势，形成产业特色，实现规模效益。

　　抓好企业改革创新。继续积极稳妥地推进国有企业改革，做到因地制宜，因企施策，改革一家，发展一家。强化企业在自主创新中的主体地位，引导创新要素向企业集中，加快建设以企业为主体、市场为导向、产学研相结合的技术创新体系。支持有条件的企业建立国家级和省级企业技术中心。大力发展高新技术产业，加强企业技术改造，突出抓好晶鑫光伏产业太阳能多晶硅、明星麻业麻类高档面料、瑞亚竹麻纺织、天心博力高品质仿金铜合金粉及金属油墨、橡机重大装备出口基地、桃江有色金属冶炼工业园、安化钨锑钒精深开发、海纳百川生物柴油产业化、东方水泥日产2500吨新型干法水泥生产线等一批重大技改项目建设，促进产业结构优化升级，全面提升产业竞争力。确保全年技改投资增长30%以上。

　　加快发展乡镇工业。因地制宜，依托资源，立足实际，突出特色，认真抓好乡镇工业基地或工业小区建设，促进乡镇工业集约集群发展。加快乡镇工业机制创新和技术创新，提升乡镇工业整体素质，促进乡镇工业总量上规模、质量上档次、管理上台阶。

大力发展循环经济。重点建设好沧水铺循环经济带，争取列入国家第二批循环经济试点计划。高明经济工业区和新河桥加工基地，要按循环经济要求，进一步完善发展规划，强化环保建设与监管，争取进入省循环经济试点项目计划。

　　重视和培养企业家。要坚持培养和引进相结合，大力培育职业经理人，不断壮大企业家队伍。大力营造有利于企业家干事创业的良好环境，关心、爱护、保护企业家，强化激励措施，对有突出贡献的企业家，在政治上给予鼓励，在经济上给予奖励，充分发挥企业家的智慧，激发企业家的创业激情。

　　（二）扎实推进社会主义新农村建设

　　紧紧抓住重大历史机遇，贯彻“工业反哺农业，城市支持农村”的方针，按照“生产发展、生活宽裕、乡风文明、村容整洁、管理民主”的要求，充分发挥农民的主体作用，突出发展农村经济，全面推进新农村建设。

　　加快发展现代农业。稳定发展粮食生产，引导支持200万亩优质稻和60万亩超级稻基地建设；按照高产、优质、高效、生态、安全的要求，引导支持蔬菜、水果、茶叶、棉花、苎麻生产，建设120万亩优质油菜、100万亩优质棉麻、100万亩蔬菜等一批无公害、绿色、有机农产品生产基地，做大做强安化黑茶产业；全面启动国家现代林业示范市建设工作；加快养殖业规模化、集约化、标准化、产业化发展步伐，大力推进养殖方式的转变，提高科学养殖水平和经济效益。引导农民发展高效园艺业。按照“扶优、扶大、扶强”的原则，培育一批科技含量高、生产规模大、竞争能力强的农产品加工龙头企业。抓好兰溪米市、益华水产加工城、益阳农产品加工基地，发展一批特色专业加工乡镇，全力推进“一村一品、一乡一业、一县一园”建设，实现现代农业与新型工业化的有效对接。提高农业市场化程度，支持发展农民专业合作组织、中介组织和购销大户，鼓励发展经纪人代理、农产品期货等新型流通方式和流通业态，建立健全农产品流通“绿色通道”，健全农业信息网络。加快种养业良种培育，抓好新技术示范和新品种推广应用，完善动植物重大疫病检测防控体系。大力推进农业标准化生产，加强农业品牌建设，提高农产品质量安全水平。

　　切实加强农村基础设施建设。高度重视防汛抗灾工作。继续抓好防洪抗旱和农田水利、农业机械化、乡村清洁工程等一批工程和项目建设。加强气象、水文监测工作，加快防灾减灾体系建设。实施农村公路通畅和通达工程，改造和新建通行政村砼路面1300公里以上。加快完善农村电网，改善农村通信条件，提高互联网进村入户率。加快农村改水、改厨、改厕、改栏，解决8万农村人口饮水安全问题，推广节柴灶5万个，改厕7万个。积极发展清洁能源，大力发展农村小水电，新建沼气池1.2万个。加大土地整理开发力度，开发总规模突破10万亩。

　　努力增加农民收入。把全面提高农民素质作为新农村建设的基础工作，加强以提高职业技能为重点的农民工培训，提高农民外出就业、稳定就业的能力，培养造就有文化、懂技术、会经营的新型农民。挖掘农业内部增收潜力。发展农村二、三产业和多种经营，加大农业旅游开发力度。大力发展劳务经济，积极拓展市外劳务市场，巩固和发展劳务品牌，建立健全保障进城务工人员合法权益的机制，全市农村劳动力转移就业突破100万人。鼓励外出务工经商人员回乡创业。认真落实国家对农民的种粮补贴、良种补贴、农机具购置补贴和燃油补贴，继续实施粮食最低收购价政策，以政策保证农民增收。切实加强涉农价费监管，进一步减轻农民负担。加强扶贫开发工作，努力实现1万贫困人口达到温饱标准。

　　继续深化农村综合改革。稳定并完善农村基本经营制度，积极稳妥地推进土地承包经营权流转，深化乡镇机构、农村义务教育、县乡财政管理体制改革，推进粮食流通体制、集体林权制度改革，完善小型农田水利设施产权制度改革。建立“三农”投入和财政支农资金稳定增长机制，扩大公共财政覆盖农村的范围。积极化解乡村债务。

　　认真抓好社会主义新农村建设的基础工作。搞好村庄布局规划和建设规划。抓好社会主义新农村示范村建设。积极推进村庄治理，继续抓好乡风文明建设，大力推进民主管理。

　　（三）着力推进城镇化进程

　　要坚持规划引导、基础先行、产业支撑，积极构建以中心城区为核心、桃江和沅江为两翼、县城为重点、中心镇为依托的城镇体系，加快推进城镇化进程，力争城镇化率提高2个百分点。

　　加快中心城区扩容提质。按照“经济繁荣、环境优美、功能齐全、特色鲜明的环省会中心城市、现代化新型工业城市、宜居山水生态文化旅游城市”的城市功能定位，认真实施“东接”战略，主动对接和融入长株潭，切实加强中心城区规划管理与建设。继续做好《益阳市城市总体规划》调整报批工作，加快土地利用总体规划修编，确保中心城区建设发展用地需求。优化规划行政许可程序，确保规划的有效实施。加强中心城区基础设施建设。突出加快“东接”基础设施建设，确保益阳至宁乡一级公路年内开工，加快省道308线西姚公路建设，争取319国道益阳南线绕城高速公路尽快开工。完成银城南路建设，加快城市防洪续建工程建设，抓好秀峰水系改造，开工建设梓山东路、迎宾东路、会龙山公园、梓山湖公园，加强城市配套设施建设。切实经营好城市，筑实城建投融资平台。加大土地经营开发力度，依法规范收费管理，拓宽筹资渠道，全面归集城建资金，建立健全城建资金筹集、使用、偿还三统一的城建投融资良性运行机制。理顺城市管理体制，着力构建城市管理长效机制。办好市政公用事业，强化市容环境卫生管理，为市民提供一个优美的人居环境。

　　加快小城镇建设。抓好县域城镇体系规划。发挥比较优势，坚持分类指导，以点带面，突出特色，加快产业聚集，深化户籍制度改革，千方百计加大投入，加快城镇基础设施建设，抓好以环境卫生为重点的各项管理，推动县城和中心镇加快发展，增强城镇的竞争力和聚集力。

　　（四）突出抓好招商引资和项目建设

　　切实抓好招商引资。强力实施招商引资目标管理，完善招商引资考核体系，力争引进投资在1亿元以上的项目20个以上。继续开展招商引资年活动，形成全方位招商，全民招商，市县联动，各部门共同参与的招商引资工作格局。认真承办“第三届湘台经贸交流与合作高峰论坛”，精心组织参加在长沙举行的“珠洽会”和省农博会、在广州举行的全国进出口商品交易会、在郑州举行的“中博会”，组织好“益阳（浙江）招商活动周”。突出引进战略投资者，突出工业园区招商，突出产业项目招商。着力整合招商资源，提高行政资源在招商引资中的运行效益。强化招商网络建设、项目的开发与包装、企业在招商引资中的主体地位和作用，进一步改进招商方式，抓好乡情招商、以商招商、网上招商、小分队招商、中介招商，增强招商引资的实际效果。进一步优化招商引资环境，努力实现招商引资质量、招商队伍建设、引进战略投资者的新突破。

　　切实抓好项目建设。完成沅江220千伏输变电工程、麻河口大桥、马奎路、沅茅路建设，加快纳爱斯二期、5万吨辣妹子生产线、益阳电厂二期、柘溪电站扩容、东坪电站、株溪口电站、白竹洲电站、赤山大桥、省道202线华茅公路、省道204线南茅公路、益阳至芦林潭航运工程建设步伐，新开工建设150万吨林浆纸首期60万吨工程、中南农机动力中心、金立平板电视制造基地、核技术产业化基地、安化石煤综合发电、中冶和紫金矿产资源综合开发、桃马公路、二广高速益阳段等项目，抓紧做好桃花江核电站、杭瑞高速益阳段等项目的前期工作。抓好电网改造、通信、电子政务等项目建设。

　　（五）加快第三产业发展

　　加快发展服务业。充分发挥现代服务业对经济发展的支撑和推动作用。着力发展现代物流、金融、商务、信息等生产性服务业，规范发展法律、会计、审计、评估、咨询、产权交易等专业服务业。鼓励发展社区服务业，改造提升传统服务业。

　　大力发展文化旅游业。把旅游业作为支柱产业来培植。扎实开展中国优秀旅游城市创建工作。完善旅游配套设施，把梓山湖国际高尔夫俱乐部、益阳奥林匹克公园等景区打造成健身休闲旅游的主导产品。高标准建设桃花江竹海风景区、安化茶马古道、南洞庭湖湿地、黄家湖生态园等旅游精品项目。不断提升农家乐系列产品的品位，使之成为全省农村休闲旅游和新农村建设有机结合的样板示范工程。充分挖掘地方文化，着力打造益阳国际茶文化节、洞庭渔火节、桃花江美人文化节等旅游节庆品牌。强化旅游宣传促销和行业管理，促进宾馆饭店上星级、旅游景区上等级、旅行社上水平、旅游产品出特色，不断壮大旅游产业规模。接待游客人次和旅游总收入分别增长20%和22%。

　　努力拓展城乡消费市场。规范发展房地产业，积极推动普通商品住房建设，完善市场化、社会化物业管理，改善城镇居民居住条件。合理引导文化、汽车、通信、健身等消费，促进消费结构升级，扩大城镇消费。继续抓好“万村千乡市场工程”，全面推进农村市场服务体系建设，着力引导和促进各类投资主体到农村投资建设商业设施，鼓励各类连锁超市向农村延伸，发展一批现代连锁农家店，规范农资流通渠道，繁荣农村市场，丰富消费种类，释放消费潜力，进一步扩大农村消费。努力优化消费环境，严厉打击损害消费者权益的行为。

　　积极扩大对外经济贸易。引导支持拥有自主知识产权的高新技术和农产品精深加工企业扩大出口规模，扶持出口龙头企业加大技改力度，提高承接加工贸易转移的能力。实现进出口总额突破2.2亿美元，增长20%以上，其中出口1.2亿美元。积极争取长沙海关在我市设立办事处。鼓励有条件的企业到境外投资，努力扩大对外劳务输出。

　　（六）抓好财税金融工作

　　加强财税征管。加大税法宣传力度，严格依法治税。加强纳税评估，坚持严征细管。抓好重点税源的征收入库，解决企业存在的实际困难，靠“扶大”增收；加大对零星税种的征管力度，靠“抓小”增收；加强对重点税源的监控和稽查，强化对建筑安装、房地产、房屋租赁税收等税收薄弱环节的征管，靠“堵漏”增收；清理和规范税收优惠政策，防止税收流失。加强国有资产监督和管理。

　　完善财政支出管理。切实保障人员经费、社保支出、维稳支出和对社会主义新农村建设的投入，加大对社会公共事业发展的投入，让广大群众共享改革发展的成果。进一步完善部门预算改革，深化非税收入管理制度改革，积极推进国库集中收付制度改革，全面落实政府收支分类改革，继续深化农村财政综合配套改革，完善财政运行机制。健全财政专项资金管理、分配、使用、拨付制度，确保专款专用。坚持勤俭过日子，节约办事业，大力压缩非发展性支出，提高财政效益。

　　引导金融机构加大对地方经济发展的支持力度。积极支持各类金融机构在益阳发展业务。支持以农村信用社为重点的各类金融机构的改革和发展。积极扩大金融开放和竞争。引导金融机构优化信贷结构，改善对企业的信贷服务，扩大信贷投放量。增强全社会的保险意识，引导保险市场健康有序发展。鼓励、支持各类信用担保机构发展，引导、扶持有条件的企业上市融资，努力实现我市上市公司和担保机构零的突破。支持加强金融监管，切实加强金融生态环境建设，维护金融稳定和安全。

四、着力构建和谐益阳

　　统筹发展各项社会事业。坚持教育事业优先发展。强化政府提供教育公共服务的职责，落实农村义务教育经费保障新机制，依法加大教育投入，全部免除农村义务教育阶段学生学杂费。继续调整和优化学校布局，扩大优质教育资源。巩固和提高“普九”成果，切实抓好“两基”迎国检工作。积极发展幼儿教育和特殊教育，稳步发展高中阶段教育和高等教育。大力发展职业教育，规范发展民办教育，支持国基职业教育基地发展。逐步建立和完善特困学生救助体系。深化教育管理体制改革。加大教师培训力度，加强师德师风建设，建立一支高素质的教师队伍。加快科技创新步伐。认真实施《益阳市科技发展第十一个五年规划纲要》，积极推进科技创新、科技支撑和科技富民强县示范工程，构建以国家、省、市级企业（工程）技术中心为骨干、产学研相结合的自主创新体系。加强科技招商，启动市生产力促进中心建设，抓好博士后工作站建设。加强科学普及，加快产业领域关键技术等科技成果的转化利用。牢固树立人才是第一资源的观念，完善人才评价、选拔任用和激励机制，大力培养和引进各类人才。完善疾病预防控制体系和突发公共卫生事件应急机制。强化职业病防治措施，切实抓好艾滋病、血吸虫病、乙型肝炎、结核病等重大疫病的防控。继续抓好乡镇卫生院的改造，普及新型农村合作医疗，大力发展社区卫生服务，加强医德医风建设，为城乡居民提供安全、有效、方便、价廉的公共卫生和基本医疗服务。大力发展妇幼卫生事业。积极创建国家卫生城市，深入开展群众性爱国卫生运动。切实抓好广播电视“村村通”工程建设，整合全市有线电视网络资源，努力扩大无线覆盖。加强广播电视媒体建设，提高自办节目质量。积极稳妥推进文化体制改革，抓好乡村网络文化信息资源共享工程建设。编印好益阳历史文化丛书，繁荣文艺创作，重视“三周”文化研究，鼓励花鼓戏等地方戏曲发展，发展群众文化社团，丰富城乡文化生活。重视文物保护，抓好非物质文化遗产保护、申报、开发和利用工作。健全全民健身服务体系。努力打造羽毛球等品牌运动项目，积极为国家培养和输送竞技体育后备人才。继续申办国际性高水平体育赛事。健全“依法管理、村（居）民自治、政策推动、优质服务、综合治理”的人口计生工作长效机制，坚决落实计划生育目标管理责任制，进一步强化流动人口计生管理和部门综合治理，稳定低生育水平，提高出生人口素质，实现进位争先目标。大力发展妇女儿童事业，保障妇女儿童合法权益。积极发展残疾人事业和社会慈善事业。抓好市博物馆、中心医院、特殊学校的二期工程和城市规划展示馆建设，启动市国家综合档案馆和市妇女儿童活动中心建设，抓好社区建设和农村敬老院的改造扩建，支持乡镇计生服务所、文化站和农村图书销售点建设。提高国防动员能力，增强全民国防意识，加强人民防空工作，深入开展“双拥”活动，巩固发展军政、军民团结，切实做好民族、宗教、外事、侨务、保密、对台、地震、老龄、地方志、资产办等工作，促进各项经济社会事业全面协调发展。

　　进一步解决群众生活中的突出问题。把就业纳入经济社会发展评价体系，努力营造全民创业氛围。进一步完善就业服务体系，建立规范的就业援助机制和统一的人力资源市场。采取过硬措施解决“零就业”家庭的就业困难问题。做好大中专毕业生就业和复转军人的安置工作。强化社会保障制度建设，努力扩大社会保险覆盖面，完善基本养老、失业、基本医疗、工伤和生育保险，发展企业补充养老保险和商业保险。健全以城乡低保、农村五保户供养和灾民救助制度为基础，以医疗、教育、住房、扶贫等多项救助制度为重点的社会救助体系，启动建立司法救助基金工作，加大法律援助力度。重视和抓好库区建设，改善移民生产生活条件。健全住房公积金制度。抓好廉租房和经济适用房建设与管理。认真执行征地拆迁政策，规范拆迁行为，确保征地补偿安置到位。建立健全最低工资和劳动保障监察制度，维护劳动者的合法权益。认真做好国有企业改制职工安置工作，落实各项政策性补偿。根据省政府的统一部署，结合我市实际，从解决人民群众最关心、最直接、最现实的利益问题入手，为人民群众办理8件实事，并采用行政问责制的办法保障工作落到实处。

　　加强社会主义民主法制建设。推进民主政治建设，认真贯彻落实《监督法》，主动接受人大及其常委会的法律监督和工作监督，坚持重大事项向人大及其常委会报告制度，认真执行人大及其常委会的决议、决定。加强与人民政协的联系与协商，自觉接受政协民主监督，积极支持政协履行职责。进一步提高人大代表议案、建议和政协委员提案的办理质量。加强与民主党派、工商联和工、青、妇等人民团体的联系，改善民主党派、工商联和人民团体的工作条件。深入实施依法治市战略，扎实抓好“五五”普法教育。

　　维护社会公平正义。依法建立以权利公平、机会公平、规则公平、分配公平为主要内容的社会公平保障体系。整顿市场经济秩序，严厉打击经济违法犯罪活动，坚决制止各类不正当竞争。理性审视经济发展过程中各方利益诉求，进一步拓宽社情民意表达渠道，建立健全社会预警、矛盾调处、权益保障等机制和制度，依法及时解决群众反映的问题。切实加强思想疏导工作，妥善处置群体性事件。认真落实社会治安综合治理责任制度，夯实基层，强化基础，完善城乡联动的治安防控体系，全面整治社会治安秩序，努力创建“平安益阳”。依法严厉打击严重刑事犯罪、“法轮功”等邪教组织和地下“六合彩”等赌博活动，坚决取缔各类非法传销组织。加强安全生产和安全管理，层层落实安全生产责任制，严格执行安全许可制度。加大安全检查执法力度，深入开展重点行业、重点领域的专项整治，严厉打击非法生产经营行为，坚决遏制重特大安全事故发生。整顿和规范医药市场秩序，加强卫生监督执法和食品、药品、餐饮卫生监督管理，确保公众饮食、用药安全。

　　推进社会主义精神文明建设。继续抓好邓小平理论和“三个代表”重要思想的学习，加强落实科学发展观和构建社会主义和谐社会重大战略思想的学习宣传，不断巩固全市人民团结奋斗的思想基础。继续抓实文明机关、文明窗口、文明社区和“十星”级农户、“五星”级居民户等文明创建活动，倡导文明健康的生活方式，提高城乡文明程度。深入开展“扫黄打非”行动，大力整治网吧，确保文化市场和出版物市场健康发展。支持《益阳日报》、益阳广播电视台等主流媒体加快发展。

　　促进人与自然和谐发展。坚持经济发展与环境保护并重，资源开发与节约并重，努力建设资源节约型、环境友好型社会。继续推广清洁生产、清洁能源和新型墙材，大力发展节地、节能、节水型经济，集约开发利用矿产资源，强化国土资源管理。严格森林、湿地、水资源保护和管理，搞好中心城区、洞庭湖区、资水流域的生态保护和生态建设，抓好创建国家森林城市工作。加强环保宣传教育和环保执法，开展环保综合整治，抓好重点区域、重点行业的污染治理。

五、加强政府自身建设

　　坚持依法行政，建设法治政府。认真贯彻实施国务院《全面推进依法行政实施纲要》，严格按照法定权限和程序行使职权，切实将政府管理经济社会行为纳入依法运转的轨道。认真落实执法责任制，完善行政复议、行政赔偿和补偿等制度。加强和改善中心城区行政综合执法工作。加强行政执法内部监督，健全行政执法过错追究制度。强化外部监督体系，构建完善、管用、有效的依法行政监督机制。

　　切实转变职能，建设服务政府。继续推进政企、政事和政府与市场中介组织分开，强化政府的社会管理和公共服务职能，为全体社会成员提供更多更好的公共服务。继续深化行政审批制度改革，减少审批事项，规范审批程序。加强政务中心建设。制定和完善突发公共事件的各类应急预案，不断提高应急管理能力，切实保障公共安全。切实加强政务、法制、市场和人文环境建设，着力营造良好的发展环境，让一切投资者、创业者在益阳能够安心创业、放心发展。

　　确保权责一致，建设责任政府。牢固树立权力就是责任、无功即过的观念，认真履行职责，真正做到权责一致，取信于民。加大督查督办力度，狠抓工作落实，进一步提高组织实施的能力，确保各项工作落到实处。积极推行行政问责制，把行政问责与行政监察、审计监督结合起来，坚决查处行政不作为、乱作为的行为。加强政府诚信建设，以政府诚信带动社会诚信。牢固树立正确的政绩观，尽职尽责做好各项工作。

　　规范行政管理，建设效能政府。进一步完善议事规则和决策程序，健全重大问题集体决策制度、专家咨询制度、法律论证制度、社会公示和听证制度以及决策责任制度，不断提高科学决策和民主决策水平。加快电子政务建设，充分利用现代信息技术改进政府管理。努力降低行政成本，把有限的财力物力用在人民群众直接受益的事业上。进一步明确政府各部门职能，加强部门协调配合。全面推行内部考核与公众评议、专家评价相结合的办法，充分发挥绩效考评的导向作用和激励约束作用，提高政府工作的整体效能。

　　努力改进作风，建设廉洁政府。坚持以人为本，执政为民，加强干部队伍作风建设，大力弘扬艰苦奋斗、求真务实的精神，提高公务员素质，做到讲实话、干实事、求实效，坚决反对弄虚作假。关心爱护基层干部，帮助基层解决实际问题。加强廉政建设，坚持从源头上预防腐败，建立健全教育、制度、监督并重的惩治和预防腐败体系，加大对工程建设、土地出让、产权交易、政府采购等领域腐败行为的查处力度。切实精简文件、会议，改进文风、会风。大力推行政务公开，完善政府新闻发布制度和信息公布制度，增加政府工作透明度，把政府及其部门的行政行为置于人民群众的监督之下，提高全市人民对政府工作的满意度和信任度。

　　各位代表，回顾过去，我们信心倍增，展望未来，我们激情满怀。让我们紧密团结在以胡锦涛同志为总书记的党中央周围，高举邓小平理论和“三个代表”重要思想伟大旗帜，在市委的坚强领导下，全面贯彻落实科学发展观，着力构建社会主义和谐社会，与时俱进，开拓创新，求真务实，奋发有为，为实现我市经济的后发赶超而努力奋斗！
